# Teaching “Shock Pathophysiology” by Flipped Classroom

**DOI:** 10.12669/pjms.35.6.1211

**Published:** 2019

**Authors:** Syeda Sadia Fatima, Satwat Hashmi, Rehana Rehman, Rozmeen Akbar

**Affiliations:** 1 Dr. Syeda Sadia Fatima, PhD. Assistant Professor, Department of Biological & Biomedical Sciences, Aga Khan University, Karachi, Pakistan; 2 Dr. Satwat Hashmi, PhD. Assistant Professor, Department of Biological & Biomedical Sciences, Aga Khan University, Karachi, Pakistan; 3 Dr. Rehana Rehman, Ph.D Physiology. Associate Professor & Vice Chair Research & Graduate Studies, Department of Biological & Biomedical Sciences, Aga Khan University, Karachi, Pakistan; 4 Dr. Rozmeen Akbar, MBBS. Teaching Associate, Department of Biological & Biomedical Sciences, Aga Khan University, Karachi, Pakistan

**Keywords:** Flipped classroom, Medical Students, Shock

## Abstract

**Objective:**

To assess usefulness of flipped style of teaching conducted as small-group format in Cardiovascular and Respiration module for Year-I undergraduate medical students at Aga Khan University.

**Methods:**

The study was planned and conducted over a period of eight months from March to October 2017 including the time taken for planning, mock run, execution followed by analysis and dissemination. It was carried out at the Aga Khan University Medical College, Karachi. Pre and post test scores of students after flipped class room sessions was compared. Moreover, perception of students was assessed on Likert scale (0-4) by a pretested validated questionnaire.

**Results:**

The mean pre-test scores of the students was 4.86 ± 0.91 which improved to 6.09 ± 0.81 (p = 0.021) after attending the flipped class session. Students approved that the frame work helped to promote their learning motivation and engagement with improvement in understanding of the course materials and enhancement of learning during Face to Face activity.

**Conclusions:**

The flipped classroom approach showed promise in teaching and learning of ‘Pathophysiology of Shock’ by clinical scenarios in small group discussions. Implementation of flipped class room activity on a wider scale however needs careful selection of course objectives and logistics.

## INTRODUCTION

Educating contemporary physicians is a challenge. Not only these ‘digital natives’ are totally in tuned to the new and evolving digital world, but their way of thinking and processing information is fundamentally different from their predecessors.^1^ To enhance learning in this modern era, new innovative learning models are continuously being developed. Flipped classroom (FCR) is one such model. It is an active learning pedagogical method in which the students prepare prior to class using different modalities, e.g. reading materials and videos and spend the time in class discussing the content and reinforcing the concepts.^2^

The goal of this ‘flip’ style of teaching is to engage students in interactive exercises to facilitate learning and in-depth understanding of concepts and enhance retention of knowledge.^3^ Flipped classroom has generated a lot of attention in medical education simply because it was found to be a better way of adult learning than traditional didactic lectures.^4^ Research showed that students were found to spend more time on average reviewing books and learning material in flipped style learning.^5^ It allowed adult learners to integrate new knowledge with exiting knowledge effectively.^6^ It was also found to be more enjoyable, and provided a positive learning experience for undergraduate and graduate level as well as for preclinical and clinical teaching alike.^7^,^8^

We at Aga Khan University follow the problem-based learning (PBL) approach in the preclinical years in the undergraduate medicine curriculum. Flipped classroom is being introduced in the curriculum as an innovative method to engage the new generation of students. The objective of our study was to assess usefulness of flipped style of teaching conducted in small-group format in Cardiovascular and Respiration module for Year-I undergraduate medical students at Aga Khan University.

## METHODS

This cross sectional survey was conducted at the Aga Khan University Medical College, Karachi in between March and October 2017, after receiving approval from the by Ethical Review Board of Aga Khan University (4667-BBS-ERC-17). The study enrolled first year undergraduate medical students who were taking the Respiration and Circulation Module at the Aga Khan University. The study spanned over a period of eight months (March-October 2017) including the time taken for planning (3 months), mock run (2 weeks), execution (2 weeks) followed by analysis and dissemination (4 months).

### Conceptual Framework of FCR

***Planning of FCR*** In order to conduct these sessions effectively; a well-versed study and teaching plan was required for which three facilitators worked together. All three facilitators were subject specialists holding PhD’s in their relevant fields. The two main categories of shock (cardiogenic and hypovolemic) were selected as a core concept of interest based on the learning objectives of the module. The subtopics or learning objectives to be covered in the flipped classroom were then divided based on the blooms taxonomy to be covered in either non-face to face (NF2F) or face to face sessions (F2F). The learning objectives selected for NF2F sessions were: a) Define shock b) Classify and give examples of the four main types of shock: hypovolemic, cardiogenic, obstructive, and distributive and c) Identify common causes for these shock types. While for the F2F session was a) Discus the pathophysiology of cardiogenic and hypovolemic shock and b) recognize the clinical and laboratory features of cardiogenic and hypovolemic shock. Furthermore, a provision of protected study time was added in the schedule so that students wishing to complete the NF2F tasks at the university could do so easily.

***Pre-Run*** Since it was decided to divide the class into 3 small groups to ensure maximum student-facilitator relationship and mastery of concept; three facilitators were engaged. These facilitators met multiple times to ensure that each and every objective was being covered, designed a map for session timings so that they were well synced and that their information was well versed.

***Non-Face to Face Component (NF2F)*** For the NF2F component video lectures freely available from Khan Academy and book chapters’ excerpts from Guyton and Hall text book of medical physiology 13^th^ edition, Sherwood textbook of physiology 9^th^ edition and Ganong textbook of physiology 25^th^ edition was given to the students. The reading material and video links were provided to students via the one 45 system (student portal), and email, one week before the session was planned. These pre-session lectures delivered key concepts via visual graphics and real-life examples for the topic being studied and the book chapters further strengthened the concepts discussed in the video lectures. To ensure student compliance with task completion they were asked to solve a quiz (pretest).

***Face to Face Component (F2F)*** The class was divided into three small groups and a facilitator for each group was assigned to help and address student queries. The suggested time frame for the class was 90 minutes, with 15 minutes for review of the instructions and division into groups, 40 minutes for the group activity, and 20 minutes to complete the post class survey.

Cases and questions were given to students to solve in groups. At the end of the F2F session, students in each group were given the task of designing a flowchart/diagram to explain the pathophysiology of shock. This strategy combined the Team Based Learning TBL style learning with the FCR pedagogy in order to enhance student knowledge, retention and understanding of concepts.

***Post-test*** A post test was conducted which helped show the overall improvement in each individual student’s knowledge after the F2F session. Questions in pre and posttest were different but centered around the concepts relevant to the topic of shock.

***Quantitative Data Collection*** The questionnaire was designed to assess the response of students. It covered the following components, (a) Strategy of FCR (16 items) (b) Effectiveness of FCR on ‘Pathophysiology of Shock (11 items) and (c) Open ended comments for additional points from the students regarding the FCR. The items (a & b) were rated on a five-point Likert scale (strongly disagree, disagree, neutral, agree, strongly agree; 0-4) pretested on a group of 10 students. It was piloted on students who did not participate in the study. The pilot testing ensured validity of tool. Cronbach’s alpha for the questionnaire was 0.88. These results suggest that the tool is valid and reliable. Immediately after the F2F all respondents were informed about the purpose of the survey and students were invited to complete the end of class questionnaire to record the learning experience.

## RESULTS

**Fig. 1:**
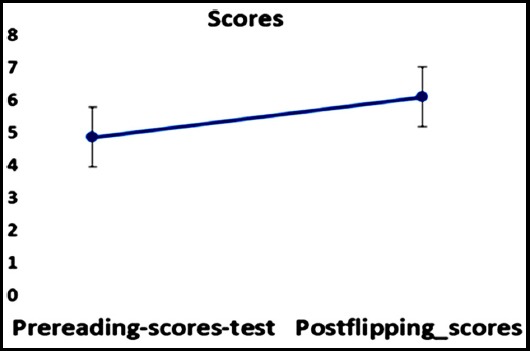
Schematic flow of events of the session from planning to post test. Overall comparison of cohort between Pre and post test scores.

The student’s demographic characteristics, post class survey response and pre and post test scores are shown in Table I and II, and Fig. 1. A total of n=40 students with a male to female ratio of 22:18 took part in the activity.

**Table I:** Student’s perception of FCR Strategy.

	Agree	No Opinion	Disagree
Placement shock FCR schedule was appropriate	38	1	1
Clear instructions provided	40	-	-
Preparation material helpful for NF2F	38	2	-
Preparation material given well ahead of time	37	3	-
Learning objectives were well defined	40	-	-
Learner activated prior knowledge elaborate FCR	39	1	-
Learner developed competence self-directed learning	38	1	1
NF2F enable learner to be self-directed	35	5	-
NF2F enhanced ability information using internet library	34	6	-
F2F enhanced ability speak front of peers	39	1	-
Critical reasoning skills developed F2F	40	-	-

**Table II:** Student’s engagement and learning ‘Effectiveness of FCR’.

	Agree	No Opinion	Disagree
Able to identify the causes shock	40	-	-
Understood pathophysiology shock	39	-	1
Able differentiate between Hypovolemic & Cardiogenic shock	40	-	-
Able differentiate between Reversible vs. irreversible shock	37	2	1
Relate the sign/symptoms/principles of management shock	38	2	-
FCR format is better	38	1	1
Format used a combination of small group discussion PBL/CBL	38	1	1
FCR will help students apply knowledge clinical practice	36	3	1
FCR will help students perform better university exams	38	2	-
Found FCR more engaging than traditional lectures	39	1	-
FCR conducted small group convenient compared large group FCR	40	-	-

### Survey results

The survey results were broadly categorized into four sub themes as (a) Student’s perception of FCR Strategy (b) Student’s engagement and learning ‘Effectiveness of FCR’ (c) Knowledge gain and (d) Open Ended Comments.

### Student’s perception of FCR Strategy

One part of the survey evaluated if placement of FCR in schedule was appropriate and pre-session content objectives meet the course outcome. Majority of students reported confidence in class planning, placement and meeting content objectives. For several of the questions, 100% of students strongly agreed that their ability to understand the learning objectives, instructions, preparation material for the NF2F component was enhanced. Based on the data, most students (97%) found the preparation materials to be helpful and the time spent for preparation to class appropriate.

### Student’s engagement and learning ‘Effectiveness of FCR’

The second part of the survey evaluated the in-class activity and ability of the students to apply the concept of cardiogenic and hypovolemic shock. More than 95% of the students reported that they were confident in identifying the causes shock, and understand the pathophysiology of shock by reading and watching the pre-session material. During the in-class activities they were able to differentiate between Hypovolemic & Cardiogenic shock based on the clinical presentations and were able to assess the severity and principles of management of shock. The FCR model was appreciated and more than 87% of the students’ responded that it should be used more often and across the teaching years in medical school. The majority of students enjoyed this small group class more than a traditional lecture. They also agreed that this method enabled self-directed learning, enhanced public speaking and critical reasoning skills as they discussed and defended their answers. This in turn helped them stay engaged throughout the session. Similarly, 87% said that they learned much better in FCR as compared to their regular classes.

### Knowledge gain

For evaluating the knowledge gain during sessions; a pre and post test was conducted and scores are shown in and **Fig. 1**. The students solved questions on the concept being taught based on the bloom’s taxonomy during the NF2F and at the end of F2F. On the average the pre-test scores for the cohort was 4.86 ± 0.915 while the post test scores for the same students improved to 6.09 ± 0.811. This led to a difference in means for the knowledge curve as 1.23 points in favor of knowledge learned.

### Open Ended Comments

The post class survey in the end also solicited open-ended comments or suggestions regarding the class. Students commented that the class was fun, interactive, and a more effective method of learning compared to an orthodox lecture. Students liked the assigned roles and the student-driven nature of the class. They stated that it was helpful to work in teams to answer questions.

## DISCUSSION

A paradigm shift in planning of curriculum from discipline-based to integrated problem-solving curriculum has revolutionized teaching and learning modalities.^9^ According to the survey data, students felt that they enjoyed the FCR more than a traditional lecture, learned more from this type of class format, and were able to utilize teamwork skills in class. When we enquired about the usefulness of this teaching learning method; a vast majority of students replied that they were able to exercise self-directed team-based learning during the small group flip session. Furthermore, they remained focused in academic group activities during the session rather than feeling bored or unable to follow the lecture. This was mainly attributed to the improved student–facilitator interaction during the case-based discussions. Rehman et al has also emphasized that comprehension of concepts with integration of mechanisms through orientation of clinical aspects augments learning in medical students.^10^

In these flipped versions, the classroom setting was more like that of team-based learning, and both students and facilitators related better to this approach. When the flipped class-room model was first piloted by our group for AKU- UGME students, similar student opinions showed a strong preference for this pedagogy.^11^ Likewise, the positive student response is consistent with work conducted across the globe.^12^-^15^ The strength of the study is utilization of small class format which encourages and stimulates collaborative team work.^16^ In the small class format, although the facilitator played an important role in enhancing the learning environment, students well prepared with the content led the group and generated the discussion with their peers to disseminate subject knowledge. An example of which is the flow charts prepared by the students.

As is evident from literature that medical educators should construct FCR model on the basis of specific content, pre-class workload suitability for students and appropriate time allocation for the flipped classroom approach.^17^ In response to comparison of FCR with other teaching modalities the facilitators as well as the leadership insisted on careful selection of topic for FCR since the model is more favorable for content that is more concrete and less abstract. We did not compare the group of students that had FCR with those that had gone through the same course without FCR; however this study establishes the usefulness of FCR as an important teaching and learning modality.

## CONCLUSION

The flipped classroom approach through clinical scenarios discussed in the form of small group discussions showed promise in teaching and learning of ‘Pathophysiology of Shock’ through integration of both NF2F and F2F components of the flipped classroom model. Implementation of flipped class room activity on a wider scale however needs careful selection of topics as far as the course objectives and logistic issues are concerned.

## Authors’ Contribution:

**SSF, RR and SH** designed the study, conducted the sessions, analyzed the data and wrote the manuscript.

**RA** assisted in session conduction; data entry and manuscript writing.

**All** authors reviewed and approved the final version of the manuscript to publication.
